# Impact of neoadjuvant chemotherapy on breast tissue density and its correlation with pathologic response: a retrospective artificial intelligence-enhanced radiological study

**DOI:** 10.1093/bjr/tqag114

**Published:** 2026-05-21

**Authors:** Filippo Pesapane, Adriana Sorce, Benedetta Di Millo, Ottavia Battaglia, Giulia Signorelli, Federica Ferrari, Luciano Mariano, Serena Carriero, Luca Nicosia, Elisabetta Maria Cristina Rossi, Luca Macarini, Gianpaolo Carrafiello, Enrico Cassano

**Affiliations:** Università degli Studi di Milano, Milan, 20122, Italy; Breast Imaging Division, IEO European Institute of Oncology IRCCS, Milan, 20141, Italy; Radiology Department, ASST Bergamo Est, Seriate, 24068, Italy; Department of Medical and Surgical Sciences, Section of Diagnostic Imaging, University of Foggia, Foggia, 71122, Italy; Postgraduation School in Radiodiagnostics, Università degli Studi di Milano, Milan, 20122, Italy; Breast Imaging Division, IEO European Institute of Oncology IRCCS, Milan, 20141, Italy; Breast Imaging Division, IEO European Institute of Oncology IRCCS, Milan, 20141, Italy; Breast Imaging Division, IEO European Institute of Oncology IRCCS, Milan, 20141, Italy; Diagnostic and Interventional Radiology Department, IRCCS Cà Granda Fondazione Ospedale Maggiore Policlinico, Università degli Studi di Milano, Milan, 20122, Italy; Breast Imaging Division, IEO European Institute of Oncology IRCCS, Milan, 20141, Italy; Breast Surgery Division, IEO European Institute of Oncology IRCCS, Milan, 20141, Italy; Department of Medical and Surgical Sciences, Section of Diagnostic Imaging, University of Foggia, Foggia, 71122, Italy; Diagnostic and Interventional Radiology Department, IRCCS Cà Granda Fondazione Ospedale Maggiore Policlinico, Università degli Studi di Milano, Milan, 20122, Italy; Breast Imaging Division, IEO European Institute of Oncology IRCCS, Milan, 20141, Italy

**Keywords:** neoadjuvant chemotherapy, breast tissue density, artificial intelligence, pathological response

## Abstract

**Objectives:**

This study evaluates the impact of neoadjuvant chemotherapy (NAT) on breast tissue density and examines the correlation between density changes and pathological response, while also assessing inter-reader agreement among radiologists and artificial intelligence (AI) tools.

**Methods:**

The study included 135 women with triple-negative and/or HER2+ invasive ductal breast cancer who underwent NAT. Digital mammography was performed pre- and post-NAT, with evaluations by radiologists of varying experience levels and comparison using an in-house developed AI tool based on AlexNet CNN architecture. Statistical analyses included Wilcoxon signed-rank test, linear regression, McNemar test, Spearman’s rank correlation, and intraclass correlation coefficients (ICC).

**Results:**

Post-NAT assessments showed a significant decrease in breast tissue density, with initial heterogeneously dense and extremely dense breasts decreasing from 32% and 15% to 18% and 7%, respectively (*P < .*003). Significant relationships were found between density reduction and age, menopausal status, and baseline density, and the reduction correlated moderately with pathological response to NAT. Intraclass correlation coefficient values reflected good agreement among experienced radiologists (0.68), fair agreement between expert and resident (0.55), poor agreement between expert and non-radiologist (0.39), and substantial agreement between the expert and AI tool (0.78).

**Conclusions:**

Neoadjuvant chemotherapy significantly reduces breast tissue density, correlating with pathological response, and AI tools provide consistent density evaluations. Artificial intelligence tools show promise in improving assessment consistency, with substantial agreement with expert radiologists. The findings highlight the need for standardized protocols and training to optimize breast density evaluations and suggest further refinement and validation of AI tools for clinical use.

**Advances in knowledge:**

This study evaluates the impact of NAT on breast tissue density while investigating its correlation with pathological response and inter-reader agreement among radiologists and AI tools. It contributes to the field by demonstrating the potential of AI to improve consistency in density evaluations and emphasizing the importance of standardized protocols and training for optimized clinical assessments.

## Introduction

Breast cancer (BC) is the most common cancer among women worldwide and a leading cause of cancer-related deaths.^1^ Recurrence and survival rates vary by BC subtype, with triple-negative BC (negative estrogen receptor [ER], progesterone receptor [PR], and Human Epidermal Growth Factor Receptor 2 [HER2]) being the most aggressive. Mammographic breast density, indicating the amount of epithelial and stromal elements in the breast, is an independent marker for predicting BC risk.^2–4^ Additionally, mammography’s effectiveness in diagnosing cancer diminishes in women with very dense breasts, making lesion identification challenging due to masking phenomena.^4^

Studies have shown that high breast density is also linked to a higher risk of cancer in the contralateral breast and early-onset breast cancer. Dense stromal tissue can induce proliferation in the breast epithelium, indicating crosstalk between stromal cells and epithelial cells.^5^ This underscores the importance of a consistent method for assessing and monitoring breast density to optimize breast cancer screening strategies.^2^^,^^4^

The American College of Radiology (ACR) introduced the BI-RADS system for categorizing mammographic density, typically determined visually.^6–9^ The 4th edition of BI-RADS categorized breast density by fibroglandular tissue percentage, while the 5th edition emphasized qualitative analysis, increasing subjectivity and variability among radiologists.^10^^,^^11^ This variability affects risk assessment accuracy and result reproducibility.^4^^,^^12^ Standardized protocols, adequate training, and intra-operator variability assessment, are crucial for enhancing consistency in breast density assessments.^11^

Artificial intelligence (AI) is increasingly used to measure breast density, improving objectivity and reproducibility.^12–15^

Given the impact of breast density on both the risk of developing breast cancer and the challenges in its detection, it was studied how breast density might affect treatment outcomes, particularly in the context of neoadjuvant chemotherapy (NAT).^16^^,^^17^ Neoadjuvant treatment with chemotherapy is widely used for triple-negative and HER2+ invasive ductal breast cancer, facilitating tumor downstaging, breast-conserving surgery, and potentially achieving a pathological complete response.^18^ Monitoring neoadjuvant treatment response is crucial for evaluating treatment effectiveness and guiding therapeutic decisions.^18^ Despite extensive research, there is not a specific biomarker that can predict appropriately the NAT response, highlighting the need for personalized oncological treatment. Skarping et al.^17^ found that extremely dense breasts before neoadjuvant treatment decrease pathological complete response chances, while Di Cosimo et al.^19^ concluded the opposite. Some studies observed breast density reduction post-neoadjuvant treatment, linked to structural changes and treatment-induced menopause.^16^^,^^19^^,^^20^

This retrospective study aims to evaluate the impact of neoadjuvant treatment on breast tissue density using mammography and investigate the correlation between density changes and pathological response. It also aims to assess the agreement among radiologists with different experience levels and between radiologists and AI software.

## Methods

### Ethics statement

The Institutional Review Board of the European Institute of Oncology (IEO) approved this retrospective study on December 19, 2023 (protocol UID: 4481).

### Population

The inclusion criteria for this study were women older than 18 years with a diagnosis triple-negative and/or HER2-positive invasive ductal breast cancer, clinical stage T1-2 N0 M0, who underwent NAT at a single institution. Patients were selected consecutively from the institutional database to minimize selection bias. Patients with multifocal, multicentric, and contralateral breast cancers, diagnosis of associated ductal carcinoma in situ (DCIS), and/or presence of chronic or psychiatric disorders were excluded from this study.

Patients were included between January 2018 and December 2023, and all imaging and clinical data were collected retrospectively from the institutional database.

The 135-patient clinical cohort analyzed in this study was reserved exclusively for the clinical pre/post-NAT analyses. No patient or examination from this cohort was included in AI model development, validation, or testing; overlap was excluded at patient level before model training.

Patients received anthracycline- and/or taxane-based chemotherapy, with the addition of HER2-targeted therapy for patients with HER2-positive disease.

### Imaging assessment and AI tool

Imaging assessments, including digital mammography, were conducted before and after the neoadjuvant therapy. Specifically, mammograms were performed using digital mammography in bilateral cranio-caudal and medio-lateral views, as well as medio-lateral-oblique views in tomosynthesis using Senographe Essential (GE Healthcare) and Hologic 3Dimensions (Hologic). While different imaging systems were used, standard acquisition protocols ensured uniform image quality. Radiologists were blinded to the mammographic unit used for each patient’s evaluation to mitigate bias. Additionally, our AI tool was trained and validated using mammograms from both systems to maintain consistency in breast density classification.

Four readers assessed the mammograms: 1 highly experienced breast radiologist (>20 years of breast imaging experience), 1 moderately experienced breast radiologist (7 years), 1 radiology resident (1 year), and 1 final-year medical student (non-radiologist). The human readers evaluated the mammograms for changes in breast tissue density using the American College of Radiology (ACR) BI-RADS breast density categories.^21^

The study employed an in-house developed AI tool designed to assess mammographic changes. The AI tool is based on a convolutional neural network (CNN) architecture, specifically adapted from the AlexNet model for feature extraction, and a U-Net architecture for segmentation. The AI tool was developed in 2023, during which AlexNet remained a well-validated architecture for breast imaging feature extraction. The decision to use AlexNet was based on its proven performance in mammographic image analysis, computational efficiency, and interpretability. To enhance accuracy, a hybrid approach was employed, integrating AlexNet for feature extraction and U-Net for segmentation.^22^ The AI-development dataset comprised 10 000 mammograms from patients not included in the present 135-patient clinical cohort. Data were split at patient level into training (*n = *8000), validation (*n = *1000), and test (*n = *1000) sets using stratification by vendor and BI-RADS density category. All 10 000 mammograms carried density labels for classification training. Because pixel-wise manual annotation of fibroglandular tissue is substantially more labor-intensive, full segmentation masks were available for a stratified random subset of 2000 mammograms (training *n = *1600; validation *n = *200; test *n = *200); this annotated subset was used for segmentation training and benchmarking. BI-RADS decision thresholds were derived on the training set, tuned on the validation set, and then locked before evaluation on the independent test set.

The original mammographic images, which varied in size, were normalized and resized to a dimension suitable for processing through the CNN. For the AlexNet model, input images were resized to 227 × 227 pixels. Prior to feeding into the network, image normalization was performed to ensure uniformity in pixel intensity across the dataset. Data augmentation was applied during training to prevent overfitting and improve model robustness.

For the segmentation task, the U-Net architecture was employed, which has proven highly effective for medical image segmentation. The network consisted of a contracting path for feature extraction and an expansive path to reconstruct the segmented regions, providing pixel-level classification. The initial layers of the network captured low-level features such as edges and textures, while deeper layers identified more complex patterns like tissue structures and densities. This approach allowed for precise delineation of fibroglandular tissue from non-fibroglandular regions. The output segmentation maps were refined using post-processing techniques, including morphological operations and conditional random fields, to ensure high accuracy in tissue boundary detection. To validate the segmentation accuracy, the model was tested on a separate subset of 2000 manually annotated mammograms. The segmentation performance was evaluated using dice similarity coefficient (DSC).

Once the breast was segmented into fibroglandular and non-fibroglandular regions, volumetric breast density was computed. The model measured the ratio of fibroglandular tissue volume to the total breast volume by integrating pixel intensities over the segmented areas. This allowed for an accurate estimation of breast density, considering both tissue volume and distribution.

The AI system classified breast density into BI-RADS categories based on the calculated volumetric density. A threshold-based algorithm, aligned with the ACR BI-RADS standards, was employed. The system mapped the calculated density values to 1 of the 4 BI-RADS density classes: (1) almost entirely fatty, (2) scattered areas of fibroglandular density, (3) heterogeneously dense, or (4) extremely dense. The thresholds were empirically defined using expert radiologist consensus on the training dataset.^21^

This combination of a deep learning-based feature extraction model (AlexNet) for initial image analysis and a U-Net for precise segmentation ensured that the AI tool could effectively support radiologists in classifying breast density and detecting subtle changes in mammographic features.

### Statistical analysis

All pre/post density-change analyses were based on the expert reader’s BI-RADS classification, prespecified as the clinical reference standard; AI output was analyzed separately in the agreement analysis.

Because BI-RADS density is a paired 4-level ordinal variable, overall pre/post changes in density distribution were assessed using the Stuart–Maxwell test for marginal homogeneity. As a sensitivity analysis, ordinal score change was also assessed with the Wilcoxon signed-rank test. To support category-specific interpretation, secondary exact McNemar tests were performed for prespecified dichotomizations: ACR C vs non-C, ACR D vs non-D, and high density (ACR C/D) vs low density (ACR A/B). Holm correction was applied for multiple comparisons.

Predictors of greater density reduction were analyzed using ordinal logistic regression, with degree of density decrease as the dependent variable (0 = no decrease, 1 = decrease by 1 BI-RADS category, 2 = decrease by 2 or more categories). Covariates were age, menopausal status, and baseline BI-RADS density category.

Association between density reduction and pathological response was assessed using Spearman’s rho with 95% CIs estimated by Fisher’s *z* transformation and exact 2-sided *P* values.

Inter-reader and AI-to-expert agreement for the 4-level BI-RADS scale were summarized using quadratic-weighted kappa as the primary ordinal agreement metric and ICC (2-way random-effects, absolute-agreement model) as a secondary summary metric, both with bootstrap 95% CIs.^23^ ICC values were interpreted according to Koo and Li:^23^ <0.50 poor, 0.50-0.75 moderate, 0.75-0.90 good, and >0.90 excellent reliability.

Descriptive statistics are reported as mean ± SD or median [IQR], as appropriate. Statistical significance was defined as *P < .*05. All analyses were performed using R software (version 4.1.0).

## Results

A total of 135 women with triple-negative and/or HER2-positive invasive ductal breast cancer were included in this retrospective radiological study (Table 1). This clinical cohort was fully independent from the AI-development dataset of 10 000 mammograms; no patient or examination from the clinical cohort was used for AI training, validation, or testing. Within the AI-development dataset, manual fibroglandular masks were available for a stratified random subset of 2000 mammograms (training *n = *1600; validation *n = *200; test *n = *200). Mean dice similarity coefficient (DSC) was 0.88 ± 0.04 in the training subset, 0.87 ± 0.03 in the validation subset, and 0.86 ± 0.04 in the independent test subset, indicating stable segmentation performance with limited overfitting.

**Table 1:** Participant demographics and baseline characteristics.

Characteristic	Total participants (*n = *135)
**Age**	
** Mean ± SD**	52 ± 11 years
** Range**	29-74 years
**Menopausal status**	
** Premenopausal**	97 (72%)
** Postmenopausal**	38 (28%)

**Figure 1:**
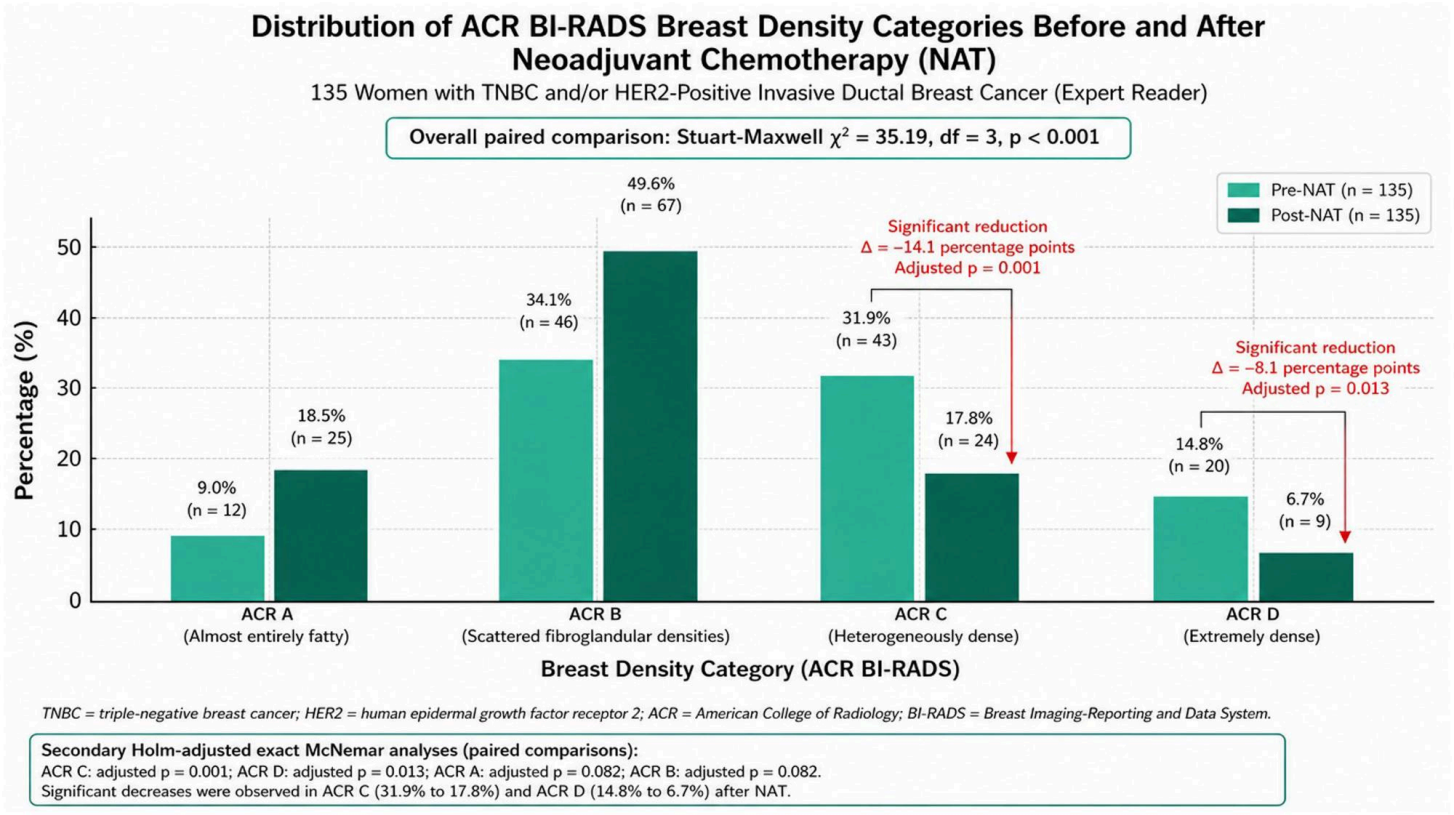
Distribution of ACR BI-RADS breast density categories before and after neoadjuvant chemotherapy in 135 women with triple-negative and/or HER2-positive invasive ductal breast cancer, based on the expert reader’s classification. The paired 4-category distribution changed significantly after NAT (Stuart–Maxwell chi-square = 35.19, df = 3, *P < *.001). Secondary Holm-adjusted exact McNemar analyses confirmed significant reductions in ACR C (31.9% to 17.8%, adjusted *P = *.001) and ACR D (14.8% to 6.7%, adjusted *P = *.013).

Using the expert reader as reference, the paired distribution of ACR density categories changed significantly after NAT (Stuart–Maxwell chi-square = 35.19, df = 3, *P < .*001; Figure 1). ACR C decreased from 43/135 (31.9%) pre-NAT to 24/135 (17.8%) post-NAT, and ACR D decreased from 20/135 (14.8%) to 9/135 (6.7%). In secondary category-specific paired analyses, both changes remained significant after Holm correction (ACR C: adjusted *P = .*001; ACR D: adjusted *P = .*013). The combined high-density group (ACR C/D) declined from 63/135 (46.7%) to 33/135 (24.4%; adjusted *P < .*001). In multivariable ordinal logistic regression, greater density reduction was independently associated with younger age (adjusted OR per 10-year increase, 0.74; 95% CI, 0.60-0.92; *P = .*006), premenopausal status (adjusted OR, 1.89; 95% CI, 1.12-3.20; *P = .*017), and higher baseline density (adjusted OR per 1 BI-RADS category, 2.41; 95% CI, 1.71-3.47; *P < .*001) (Table 2).

**Table 2:** Multivariable ordinal logistic regression analysis of factors associated with greater reduction in BI-RADS breast density category after neoadjuvant chemotherapy.

Variable	Adjusted OR for greater density reduction	95% CI	*P* value
**Age (per 10-year increase)**	0.74	0.60-0.92	.006
**Premenopausal vs. postmenopausal**	1.89	1.12-3.20	.017
**Baseline BI-RADS category (per 1-category increase)**	2.41	1.71-3.47	<.001

Notably, the relative percentage of patients in ACR A and ACR B categories increased post-NAT. This shift does not indicate an actual increase in breast density but rather reflects a redistribution effect, where many patients initially classified as ACR C or D moved into lower-density categories. Patients who were ACR A or B at baseline remained largely stable in their density classification, as their density was already low before treatment.

Density reduction correlated with pathological response (Spearman rho = 0.53; 95% CI, 0.40-0.64; *P* = 3.8 × 10^−11^), indicating that larger decreases in density tended to occur in patients with more favorable pathological outcomes.

Agreement metrics showed a clear dependence on reader expertise (Table 3). According to Koo and Li^23^ criteria, ICC-based reliability was moderate between the expert and the moderately experienced radiologist (ICC = 0.68; 95% CI, 0.59-0.76), moderate between the expert and the resident (ICC = 0.55; 95% CI, 0.44-0.65), poor between the expert and the non-radiologist (ICC = 0.39; 95% CI, 0.27-0.51), and good between the expert and the AI tool (ICC = 0.78; 95% CI, 0.70-0.85). Quadratic-weighted kappa values showed the same ranking of agreement. Descriptive statistics summarized in Table 4 show the average breast density scores pre- and post-NAT, emphasizing the overall reduction in breast density following treatment.

**Table 3:** Intra-class correlation coefficient (ICC) for inter-reader variability.

Comparison	Quadratic-weighted kappa (95% CI)	ICC (95% CI)	ICC interpretation
**Expert vs moderately experienced radiologist**	0.66 (0.58-0.74)	0.68 (0.59-0.76)	Moderate
**Expert vs resident radiologist**	0.53 (0.42-0.63)	0.55 (0.44-0.65)	Moderate
**Expert vs non-radiologist reader**	0.36 (0.24-0.48)	0.39 (0.27-0.51)	Poor
**Expert vs in-house AI tool**	0.75 (0.67-0.82)	0.78 (0.70-0.85)	Good

**Table 4:** Descriptive statistics for breast density scores.

Statistic	Pre-NAT BI-RADS category	Post-NAT BI-RADS category
**Mean ± SD**	2.50 ± 0.88	2.07 ± 0.83
**Median [IQR]**	2 [2-3]	2 [2-2]
**High density (ACR C/D), *n* (%)**	63 (46.7%)	33 (24.4%)

To enhance model transparency, the feature extraction analysis was performed by examining the convolutional layers of the AlexNet-based model, ensuring that textural and structural characteristics informed density classification. To validate the reliability of AI assessments, we compared AI-predicted BI-RADS categories with expert radiologists’ classifications, demonstrating a good agreement (ICC = 0.78).

## Discussion

Our study highlights a significant reduction in breast density following NAT, particularly in patients with high baseline breast density (ACR C-D). This finding aligns with previous reports indicating that dense breast tissue is more susceptible to chemotherapy-induced changes due to its higher stromal content. The observed reduction in ACR C-D density is clinically relevant, as high breast density is associated with increased breast cancer risk and reduced sensitivity of mammography.^19^

The increase in the proportion of patients classified as ACR A or B post-treatment does not suggest worsening prognosis. Instead, it reflects a natural redistribution effect, where many ACR C-D patients transitioned to lower-density categories after NAT. Patients already in ACR A-B before treatment exhibited minimal density changes, which is expected given their low baseline fibroglandular content.

These findings indicate that density reduction after NAT is a candidate imaging correlate of treatment effect rather than a validated predictive biomarker. Given the retrospective, single-center design and the lack of external validation, predictive and surrogate-endpoint interpretations should be avoided at this stage. The transition from ACR C-D to lower categories may improve mammographic tumor detection post-treatment, potentially aiding in follow-up evaluations and surgical planning. Particularly, decreased density may lead to improved mammographic sensitivity, enhancing the detection of residual or recurrent disease in follow-up imaging; this may also assist in surgical decision-making, especially for breast-conserving approaches, by facilitating better visualization of post-treatment parenchyma. Moreover, in patients with initially high-density breasts, the observed reduction may reflect treatment-induced stromal and glandular atrophy, which could be explored as an imaging biomarker of response.

We also found that younger women and those in the perimenopausal stage exhibited the most notable alterations in breast density after undergoing NAT. The decreased mammographic density in pre-menopausal women during chemotherapy might be attributed to induced amenorrhea, leading to lobular atrophy.^15^

Our results show that changes in breast density can be quantified reproducibly and may have exploratory value for response monitoring, but prospective validation is required before clinical biomarker claims can be made. The moderate agreement among human readers and the good agreement between the expert reader and the AI tool underline the potential for integrating AI into routine assessments to improve the accuracy and reproducibility of breast density evaluations. Artificial intelligence applied to imaging may also predict tumor response before NAT begins. A proof-of-concept study by Skarping et al.^17^ used a deep learning model with baseline digital mammograms to predict responses to neoadjuvant therapy, achieving an AUC of 0.71. This model made predictions by analyzing patterns in breast tissue and tumor appearances based on grey-level pixel variations in digital mammography.^17^

Automated assessment methods have been developed, demonstrating better correlation with human assessment and for predicting breast cancer risk, especially when using volumetric breast density.^11^^,^^24^ In 2011, Ciatto et al.^25^ demonstrated high reproducibility in the visual classification of breast density; comparing visual assessment with a computerized tool (QUANTRA) that also showed high reproducibility. Brandt et al.^15^ found moderate concordance between BI-RADS classification and 2 automated assessment tools. Youk et al.^12^ observed variable agreement in density categorization compared to visual assessment.

In 2016, Kang et al.^11^ investigated the reliability of computer-assisted methods. They compared the reliability of 3 types of computer-assisted methods for estimating breast percentage density: interactive thresholding, semi-automated, and fully automated methods. One interesting finding was the application of the semi-automated method, which focused on determining the threshold between the dense and fatty regions using automated window width and level settings.^11^ Even fully automated methods had excellent reliability in percentage density estimation, providing reliable estimates nearly free of within- and between-reader variability and reader-dependent systematic bias.^11^

Our study also utilized an in-house developed AI tool that uses machine learning algorithms to perform automated measurements, providing an independent assessment that can be compared with radiologists’ evaluations, and helping to improve consistency across different assessment methods.

Table 2 shows the different levels of agreement among radiologists and between radiologists and AI tools, underscoring the challenges and potential of integrating AI into clinical practice for breast density evaluation. The agreement was good (ICC = 0.68) between the expert and the moderately experienced radiologist, suggesting that experience in breast imaging plays a critical role in achieving consistent evaluations. Agreement was also moderate (ICC = 0.55) between the expert and the resident, and poor (ICC = 0.39) between the expert and the non-radiologist. These findings highlight the significant variability that can occur with less experienced or untrained evaluators and reinforce the need for robust training programs and standardized protocols to improve consistency among less experienced readers.^26^^,^^27^ The good agreement between the expert radiologist and the AI tool demonstrates the potential of AI to enhance the reliability of breast density evaluations. Artificial intelligence tools are not subject to fatigue or subjective interpretation, can provide standardized and repeatable assessments, thereby reducing inter- and intra-observer variability.^27^ This is particularly relevant in the context of breast density evaluation, where consistent assessments are critical for accurate risk stratification and personalized screening and treatment planning.^28^^,^^29^ Consistent experience-dependent variability has also been reported in other multi-reader breast imaging studies, further supporting the importance of reader training across modalities.^30^

One major challenge in integrating AI into radiology is ensuring transparency and interpretability of deep learning models. To address this, we incorporated explainability techniques, including feature extraction analysis, to highlight the model’s decision-making process. Our results confirm that the AI tool predominantly focused on fibroglandular tissue regions when assessing breast density, supporting its validity as a radiological adjunct. Moreover, the strong agreement with expert radiologists further reinforces the reliability of our AI-based density classification system.

Overall, our findings suggest that integrating AI into breast density evaluation can significantly enhance the consistency and reliability of assessments. However, to maximize the benefits of AI, it is crucial to ensure that human evaluators, particularly those with less experience, receive adequate training and follow standardized protocols. Future research should focus on further refining AI algorithms and exploring their integration into clinical workflows to support radiologists in providing the highest standard of care, while also addressing data availability, privacy, and generalizability issues that remain central to the ongoing evolution of AI in breast imaging.^31^ Additionally, long-term studies are needed to evaluate the impact of AI-assisted assessments on clinical outcomes and patient management in breast cancer screening and treatment.

Our study focused on assessing the agreement of BI-RADS breast density classification among radiologists with different experience levels and between radiologists and an AI tool. While we acknowledge that volumetric breast density measurements offer a more objective assessment, our goal was to evaluate the accuracy and reproducibility of BI-RADS-based assessments, which remain the standard method in clinical practice: BI-RADS classification is widely used due to its availability, ease of implementation, and compatibility with routine mammographic reporting, making our findings broadly applicable across different healthcare settings. Future studies should explore how integrating volumetric assessments with qualitative BI-RADS classification could enhance breast density evaluation and predictive modeling in breast cancer care.

Limitations of the study include its retrospective and single-center nature, which may limit the generalizability of the findings to other populations. Furthermore, the study did not evaluate the long-term impact of neoadjuvant treatment on breast density, and it lacked a control group, making it challenging to determine whether the observed decrease in breast density was solely due to neoadjuvant treatment. Another limitation of this study is the lack of external validation for the AI tool. Although the model was trained and validated on a large institutional dataset (*n = *10 000 mammograms) and demonstrated good agreement with expert radiologists (ICC = 0.78), it has not been tested on independent datasets from other institutions. External validation is crucial to ensure the generalizability of AI-driven breast density assessments across different populations, imaging protocols, and healthcare settings.

To overcome these limitations, future studies could use a prospective and multi-centric design to minimize selection and information biases. In addition, long-term follow-up could be incorporated to evaluate the impact of neoadjuvant treatment on breast density over time.

## Conclusion

This retrospective single-center study suggests that NAT is associated with reduced mammographic breast density and that larger density reductions accompany more favorable pathological response. Reader agreement in density classification was strongly dependent on experience, whereas the AI tool showed good agreement with the expert reader. These findings are hypothesis-generating and require prospective, externally validated studies before density change can be considered a biomarker or surrogate endpoint for NAT response.
